# Neuromodulation’s Role in Functional Restoration in Paraplegic and Quadriplegic Patients

**DOI:** 10.3390/biomedicines12040720

**Published:** 2024-03-24

**Authors:** Alaa Abd-Elsayed, Christopher L. Robinson, Peter Shehata, Yerin Koh, Milan Patel, Kenneth J. Fiala

**Affiliations:** 1 Department of Anesthesiology, University of Wisconsin School of Medicine and Public Health, Madison, WI 53792, USA; shehatp@ccf.org (P.S.); kohy@ccf.org (Y.K.); mapatel5@wisc.edu (M.P.); kfiala2@wisc.edu (K.J.F.); 2 Department of Anesthesiology, Critical Care, and Pain Medicine, Harvard Medical School, Boston, MA 02115, USA; christopherrobinsonmdphd@outlook.com

**Keywords:** paraplegia, quadriplegia, neuromodulation, spinal cord stimulation, spinal cord injury, functional restoration, neuroplasticity, surgical interventions, non-surgical approaches, sensorimotor function, functional rehabilitation, neurorehabilitation, electrical stimulation, motor function recovery

## Abstract

Neuromodulation is an alternative, minimally invasive treatment option that, at times, is used as a last resort for chronic pain conditions that are often refractory to other treatment modalities. Moreover, it offers promising prospects for individuals grappling with the formidable challenges posed by paraplegia and quadriplegia resulting from spinal cord injuries. This review article provides a comprehensive assessment of current treatment modalities specifically tailored for paraplegic and quadriplegic patients. We aim to evaluate the existing surgical and non-surgical interventions while delving into the role of neuromodulation in the restoration of function for individuals afflicted with these debilitating conditions. Additionally, we review the efficacy, limitations, and comparative outcomes of diverse treatment strategies available for the management of paraplegia and quadriplegia. Emphasizing the critical need for effective interventions beyond the initial 24 h surgical window, we elucidate the challenges associated with conventional therapies and their limited success in achieving comprehensive functional restoration. Central to this review is an in-depth exploration of neuromodulation’s transformative potential in ameliorating the deficits caused by spinal cord injuries. With a particular focus on spinal cord stimulation (SCS), we analyze and compare the outcomes of neuromodulation modalities and traditional treatment regimens, shedding light on the promising strides made in fostering sensory perception, motor function, and patient satisfaction.

## 1. Introduction

Paraplegia and quadriplegia are debilitating conditions resulting from spinal cord injuries (SCIs), presenting distinct patterns of paralysis and functional impairments. Paraplegia involves the partial or complete loss of motor and sensory functions in the lower extremities and trunk. Quadriplegia, also known as tetraplegia, encompasses more extensive paralysis affecting both upper and lower extremities, along with the trunk and potentially respiratory muscles [1]. These incapacitating conditions profoundly affect the individuals’ mobility, independence, and overall quality of life. Paraplegia and quadriplegia are significant hindrances to an individual’s outlook on life, potentially leading to negative effects on mental health. Current management strategies encompass a spectrum of interventions, ranging from surgical interventions targeting acute spinal cord damage to non-surgical rehabilitative approaches aimed at fostering recovery and adaptation to residual function. These conventional approaches to rehabilitate and restore functionality in paraplegic and quadriplegic patients have shown limited success, warranting exploration into innovative therapeutic avenues. The desire for newer techniques has been rapidly growing with newer technological advancements in medicine.

Currently, spinal cord stimulation is FDA-approved for primary treatment and indication of neuropathic limb pain that is resistant to more conservative therapeutic options, which will be outlined later in this article. The disorders that fall under the category of neuropathic limb pain include traditional neuropathic pain, post-surgical, post-amputation, osteodegenerative, and pain related to vascular disease [2]. Spinal cord injury patients would fall under FDA approval for spinal cord stimulation usage, as other therapeutic options provide minimal to no relief in patients, providing only a temporary solution to the lifelong pain individuals face. Also of note, neuromodulation and neurostimulation are two separate terms and should be used in different cases. Neuromodulation involves identifying neuropathic pain and intervening to provide a therapeutic response, while neurostimulation is a device that provides stimulation via inducing electrical impulses through the spinal cord to provide a therapeutic response. Furthermore, neuromodulation can consist of stimulating the spinal column to produce a biological response in addition to the application of pharmaceutical agents in small doses at the site of interest. However, neurostimulation is a form of neuromodulation, but it approaches intervention through electrical pulses induced by devices. Spinal cord stimulation would fall under neurostimulation, as leads are placed along the spinal cord of patients, sending electrical impulses throughout the spinal cord and body [3]. 

Amidst these challenges, neuromodulation has emerged as a promising frontier in medical intervention, offering a transformative approach to the restoration of sensorimotor function for individuals afflicted by paraplegia and quadriplegia. Neuromodulation has been around for many decades, with the earliest documented use of electrical stimulation beginning in 1874. Eventually, neuromodulation grew in advancement and variety to the point where, in 1968, Dr. Norman Shealy began to use dorsal column spinal cord stimulation for pain management. Additionally, around that time, in 1965, Melzack and Wall’s “gate theory” idea was presented to the world and led to the commercialization of implantable stimulators in humans [4]. Neuromodulation techniques, notably spinal cord stimulation (SCS) and other emerging modalities aim to modulate neural circuits and harness the potential of neuroplasticity to restore lost function (Taylor et al., 2022) [5].

This review article critically assesses the role of neuromodulation in the context of functional restoration in paraplegic and quadriplegic patients. We delve into the current landscape of surgical and non-surgical interventions, evaluating their efficacy and limitations in comparison to the burgeoning field of neuromodulation. By synthesizing findings from clinical studies and advancements in neuromodulation technologies, this review aims to elucidate the transformative potential of these interventions in ameliorating the functional deficits resulting from spinal cord injuries. The extent of scientific literature on neuromodulation grows each day, but in regard to spinal cord injuries, neuromodulation is a very modern technique. Neuromodulation as a safe and promising alternative has not been integrated into mainstream medical interventions; however, testing and research are underway, with positive results that inform our next steps when combating functional restoration in SCI patients. 

Understanding the evolving role of neuromodulation in facilitating functional recovery holds promise in reshaping rehabilitation paradigms, offering hope and improved outcomes for individuals enduring the challenges of paraplegia and quadriplegia following spinal cord injuries. The use of neuromodulation has the potential to change lives when it comes to ability to perform daily tasks and maintain the lifestyle individuals had before their spinal cord injuries. 

## 2. Methods

This is a narrative review article delving deeper into the potential impact of neuromodulation’s role in functional restoration in the context of paraplegic and quadriplegic individuals. The methodology behind the article selection and information processing during this narrative review began with the relevancy of the information. The articles chosen reflect the current state of neuromodulation and possess the most updated information concerning the goals of this narrative review. Additionally, the mentioned articles were chosen to display the variety of techniques used when dealing with spinal cord injuries in the immediate through long-term stages. The articles showcase both the current applications of surgical and non-surgical intervention while subsequently leading towards the emergence of neuromodulation’s application. Lastly, the articles were chosen from reputable sources and brought together to form a cohesive narrative review bringing to light the potential benefits of neuromodulation in providing new levels of functional restoration to paraplegic and quadriplegic individuals. 

## 3. Definitions of Paraplegia and Quadriplegia

**Figure 1:**
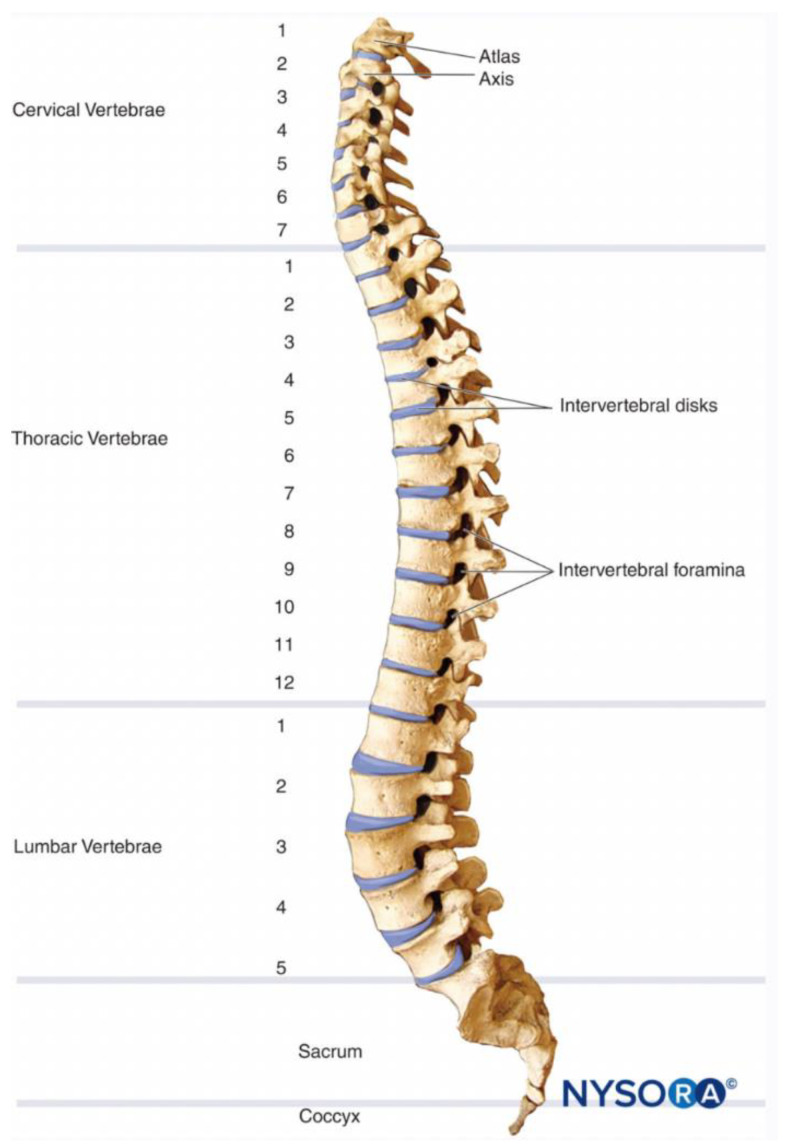
Spinal cord levels dissected into the cervical, thoracic, and lumbar vertebrae [9].

Traumatic spinal cord injuries, often caused by accidents or falls, are the leading etiological factors behind these conditions. Paraplegia and quadriplegia manifest as varying degrees of paralysis. The injury site determining paraplegia typically ranges from the C1 to T1 spinal cord segments, potentially affecting sensation throughout the entire body. In contrast, paraplegia may result from injuries occurring between the T2 and S5 sections of the spinal cord, primarily impacting sensation in the lower limbs [1,6,7] (Figure 1). Paraplegia denotes the paralysis of the lower body and legs, while quadriplegia signifies the more extensive paralysis affecting all limbs and bodily functions [7]. Spinal cord injuries leading to paraplegia or quadriplegia present with multifaceted symptoms, including loss of movement, altered sensation, and neuropathic pain [8]. These conditions significantly affect individuals’ routines and mental well-being, particularly during the transition from an active lifestyle to a more sedentary one.

## 4. Epidemiology

Prevalence rates of paraplegia and quadriplegia vary based on geographical, socioeconomic, and demographic factors. According to the NSCISC, the prevalence of paraplegia in the United States ranges from approximately 50 cases per 100,000 population, whereas complete paralysis or quadriplegia is estimated to be around 20 cases per 100,000 population [6]. Additionally, there are 17,000 new SCI cases in the United States each year [10], highlighting why making newer techniques for paraplegic and quadriplegic patients should become more of a priority. The most common neurological category of spinal cord injury is incomplete quadriplegia, covering 47.1% since 2015. This is more than double the next closest category, which is incomplete paraplegia, at 20.1%. The age group with the highest risk of spinal cord injuries is 16−30 years old [10]. However, the average at the event of the injury has increased from 29 in the 1970s to 43 years of age, as of 2015 [11]. The increase in average age at the time of the injury could be associated with better medical care or more cautious behaviors in the younger population. Furthermore, approximately 79% of new spinal cord injuries since 2015 have occurred in males. With males occupying the overwhelming majority of this statistic, this could lend itself to males engaging in more risky behavior in everyday life. From a demographic perspective, the highest percentage of spinal cord injuries at 56.1% are Non-Hispanic White Americans. However, 25% of spinal cord injuries come from non-Hispanic Black Americans, who make up 13% of the general population. The main leading causes of spinal injuries are vehicle crashes, with falls, violence, and sports following behind [11]. Identifying new solutions to better manage patient outcomes for an increasing population is of significant importance, as this is an alarmingly young group. 

## 5. Treatment Options

Treatment options for spinal cord injuries encompass both surgical and non-surgical approaches. Surgical interventions, primarily within the initial 24 h post-injury window, aim to decompress the spinal cord and, if necessary, stabilize vertebral fragments [12]. Non-surgical treatments post-surgery include physical and occupational therapy, medication, medical devices, and adapted sports, with patients primarily relying on neuroplasticity for rehabilitation. The current medical advancements have allowed for post-operative care to be shortened since the 1970s. Current lengths of stay in acute care units at hospitals have declined from 24 to 12 days on average spanning from the 1970s to 2015. Additionally, rehabilitation lengths of stay have also declined from 98 to 31 days over the same period [11]. Surgical versus non-surgical interventions target different aspects of a spinal cord injury. The 24 h window is a critical period, and surgical intervention is primarily used to prevent further damage to the spinal cord. Non-surgical techniques are geared beyond the initial 24 h and focus on providing longer-lasting relief, and, although the average time spent in the hospital decreasing indicates a level of success with current procedures, improving surgical and non-surgical techniques is still a priority. 

## 6. Surgical Interventions

Surgical interventions for paraplegia and quadriplegia post-spinal cord injury play a critical role in minimizing further damage and stabilizing the affected region. When assessing the damage to the spinal cord, there are two components to evaluate. The components include displacement and stability. Displacement accounts for the location of the individual parts of the spinal column in the form of vertebrae or discs [13]. Oftentimes, decompressive surgeries are used to prevent further damage if the orientation of the spinal cord, if left in the current position, is causing pain or will cause further damage if left untreated [13]. Decompressive surgeries, such as laminectomy, aim to alleviate pressure on the spinal cord. Additionally, stabilization involves the movement caused by damage to the spinal cord of vertebrae and discs. Stabilization procedures aim to reposition aspects of the spinal cord to minimize damage by aligning the spinal cord in the most stable position [13]. While stabilization procedures involve spinal fusion or instrumentation, these procedures aim to stabilize the spinal column after injury [14]. Fusion involves joining two or more vertebrae using bone grafts, while instrumentation involves the use of rods, screws, or plates to maintain the alignment and stability of the spinal column. The stabilization procedures help in preventing further misalignment or damage to the spinal cord [14]. Surgical options remain limited, primarily serving as preventive measures rather than reversible interventions once spinal cord damage occurs [15]. Surgical timeliness is pivotal; however, interventions beyond 24 h post-injury carry heightened risks of further spinal cord damage [15]. 

Furthermore, surgical interventions carry inherent risks, such as infection, bleeding, and neurological deficits. Patients undergoing these procedures require extensive post-operative care and rehabilitation to optimize functional outcomes and minimize complications [15]. Emerging surgical techniques like microsurgery and minimally invasive approaches show promise in reducing surgical trauma and complications, potentially enhancing recovery and post-operative rehabilitation [12,16].

## 7. Non-Surgical Approaches

Non-surgical approaches encompass an array of rehabilitative strategies aimed at optimizing functionality and improving quality of life post-injury. Physical therapy, occupational therapy, and assistive devices form the cornerstone of non-surgical management, focusing on enhancing neuroplasticity and facilitating adaptation to residual function [16]. Non-surgical approaches have been rapidly improving to provide increasingly positive outlooks for spinal cord injury patients post-surgical intervention. Pharmacological interventions, including medications for pain management and spasticity, are also integral components of non-surgical care [17]. Despite limitations to achieving full recovery, some degree of functional restoration is feasible for incomplete injuries.

### 7.1. Physical Therapy

Physical therapy plays a crucial role in the non-surgical management of paraplegia and quadriplegia. It involves various exercises and activities to maintain joint mobility, strengthen muscles, and improve balance and coordination. Without physical therapy, consequences at the musculoskeletal level will occur. Among the most significant consequences are muscular atrophy, osteopenia/osteoporosis, hypertonia, and overall restrictions of joint mobility [18]. Physical therapists tailor specific programs based on individual needs to enhance neuroplasticity and functional independence [16]. 

### 7.2. Occupational Therapy

Occupational therapy focuses on enhancing the ability to perform daily activities. It involves teaching adaptive techniques, using assistive devices, and modifying the environment to facilitate independence in activities of daily living [16]. An example of an adaptive technique is altering the sleeping position of a quadriplegic individual to prevent elbow flexion. Elbow flexion is the elbow being flexed by the individual due to paralysis in the tricep muscles. An occupational therapist would then work with the individual to implement appropriate positioning programs [19]. Occupational therapists work closely with patients to regain skills and maximize functional abilities, such as sleeping and other daily activities [16].

### 7.3. Pharmacological 

Pharmacological interventions, including medications for pain management, spasticity, and neuropathic pain, form an integral part of non-surgical care. These medications, including baclofen and gabapentin, aim to alleviate pain, reduce muscle spasticity, and manage other symptoms associated with spinal cord injuries [17], offering additional support to both surgical and non-surgical interventions by providing pain alleviation over an extended period and improving quality of life [20]. These medications serve as an additional aid to spinal cord injury patients, as the pain is ongoing well after surgical and non-surgical intervention. Pharmacological interventions are mainly used as support to the ongoing rehabilitation process, and monitoring patient usage of these medications is also a necessary component of creating a successful rehab plan. 

### 7.4. Assistive Devices and Others

Assistive devices, such as wheelchairs, braces, and orthoses, aid in mobility and provide support to individuals with paraplegia or quadriplegia. These devices assist in daily activities, enhance mobility, and promote independent living. These devices help to provide physical support for daily activities and increase patient satisfaction after a spinal cord injury. Innovative non-surgical modalities, including robotic-assisted therapies and virtual reality-based interventions, are emerging as adjuncts to traditional rehabilitation, offering novel ways to stimulate neural pathways and enhance motor recovery [16].

Despite these approaches, complete functional recovery remains a challenge, particularly in cases of severe spinal cord injuries. Completely severed sections of the spinal cord are unable to regain functionality; however, if the spinal cord is still intact through a combination of these interventions, individuals can often achieve significant improvements in functional capacity and quality of life [16]. Using a combination of surgical, non-surgical, and pharmacological interventions, the patient can experience greater functionality and pain relief, and, although restoring functionality for paraplegia and quadriplegic patients is still a challenge, neuromodulation can help. 

### 7.5. Adapted Sport

Adapted sports also serve as a great rehabilitation method for incorporating more functional restoration and granting the individual a more optimistic view of life. Adapted sports lead to spinal cord injury patients having more social participation and a greater quality of life. Go-carting has proven able to reduce muscle spasticity and provide temporary muscle relief in paraplegic individuals; however, this only lasted a couple of weeks upon stopping go-carting as a regularly scheduled activity [21]. Additionally, wheelchair-basketball has improved shoulder range of motion and overall greater functional ability when compared to a control group [22]. In multiple other categories comparing functional abilities, wheelchair-basketball was shown to increase overall physical ability in a more pronounced manner. Adapted sport and therapeutic exercise are feasible options for minimal or temporary relief and functional restoration; however, there is also a need to develop a standardized set of exercises tailored to specific individualistic needs. These workout routines would be designed to allow wheelchair users to gain maximum benefit from their adapted sport of choice while promoting social inclusion and greater life satisfaction [22]. It is important to note that, although yoga, pilates, wheelchair sports, go-carting, and climbing have been linked to providing spasticity when combating neurological disease, there is not sufficient evidence regarding the neurophysiological mechanisms [21].

## 8. Functional Restoration and Patient Selection Methods

In recent decades, models and algorithms have been created in an attempt to predict the prognosis of walking recovery. Neuromodulation could be used with these models to assess the best candidates with the greatest chance of achieving maximized functional recovery. There have been many studies conducted over the years examining motor scores and capabilities in paraplegic and quadriplegic individuals. Previous studies have focused on extremity strengths over time from visit to visit; however, in 2010 Björn Zörner developed an algorithm based on outcome predictors. With the outcome predictors, the algorithm aimed to identify sub-groups of patients with incomplete paraplegia and quadriplegia who could achieve functional walking. When looking at patients with incomplete paraplegia, the lower extremity scores and age were the best predictors of walking recovery. However, for incomplete quadriplegia, the tibial SSEP score and AIS grade were indicative of walking recovery. SSEPs, or somatosensory evoked potentials, will tell us the degree of damage to the central nervous system, while the AIS (Asia Impairment Scale) will also give us insight into the level of damage. 

Additionally, another algorithm developed as well a year later by Joost J Van. Middlethorp produced a clinical prediction rule on age and the motor scores of specific muscles. The muscles of interest were the quadriceps femurs (L3), gastrocsoleus (S1), and light touch sensation dermatomes (L3 and S1). With this prediction, great success was achieved in being able to identify patients who could walk independently, with or without braces and orthoses for <10 m [16]. These prediction models with neuromodulation could be a more promising solution for restoring functionality to paraplegic and quadriplegic patients if identifying the proper patients is achieved correctly. 

## 9. Role of Neuromodulation in Functional Restoration

Neuromodulation offers an alternative, promising avenue for functional restoration, manipulating the nervous system to potentially restore functionality to inactive nerves [23]. In combination with the studies involving algorithms, as previously stated, spinal cord stimulation in the right patients could show a great success. Spinal cord stimulation (SCS), a key neuromodulation modality, involves implanting devices into patients to deliver electrical impulses that modulate neural pathways to potentially restore lost function, enhancing sensorimotor function and surpassing conventional surgical and non-surgical interventions [23]. Spinal cord stimulation may offer long-term relief, possibly serving as the primary form of intervention when addressing spinal cord injuries regardless of severity.

Spinal cord stimulators are composed of three parts. These parts include the electrodes/leads, which come in a cylindrical or paddle form, the implantable pulse generator (IPG) or batteries, and then the charging and reprogramming equipment. The charging and reprogramming equipment comes in the form of a remote control. The spinal cord stimulator being remote controlled allows the individual to provide care for themselves without the supervision of another individual [5]. 

There are two types of spinal cord stimulators when deciding to use neuromodulation for spinal cord injuries. The first option is the SCS percutaneous trial, which involves placement of the leads in the desired area of the spine, and then removing the epidural needle. The other portion of the lead is connected to the external generator, which is then secured to the patient’s skin on the exterior. Using a percutaneous trial is better for monitoring efficacy and durability, as well as decreasing the chance of infection. The permanent trial is then used if the first use of the SCS was successful, in which the same procedure would occur but the IPG could be placed inside the skin by creating a pocket [5]. 

The mechanism of action of SCS remains to be elucidated, but preliminary murine studies suggest that neuromodulation increases the excitability of the neuronal tissue at the location of spinal injury [23,24]. In regular pattern stimulation with fixed periods (synchronized) of no stimulation of the central pattern generators (CPG), a spinal circuit involves the coordination of rhythmic activity and control of spinal reflexes, resulting in induction of adaptive plasticity; however, when stimulated randomly, it results in maladaptive plasticity, leading to increased nociceptive signaling [23,25,26,27]. When paired with physical therapy, synchronized stimulation of the spinal cord can lead to remodeling of intraspinal and supraspinal pathways, which can result in the ability to produce voluntary movement [24]. Non-surgical intervention and spinal cord stimulation pair together to give individuals the best chance at regaining functional capabilities. Of note, the spinal cord must not be completely severed in order for the stimulation to function properly [23]. This allows for plasticity to be an option, as a completely severed spinal cord has no chance of functional restoration. 

Neuromodulation’s ability to augment sensorimotor control and facilitate daily activities positions it at the forefront of spinal cord injury management, holding promise for enhanced functional restoration [23]. SCS has also demonstrated promising outcomes in reducing pain and improving quality of life for individuals with spinal cord injuries [23]. Additionally, emerging technologies in neuromodulation, such as epidural stimulation and brain–computer interfaces, show potential for further functional restoration and improved mobility [28]. 

With epidural electrical stimulation (EES), the brain can exploit functionally silenced pathways to produce movement in otherwise paralyzed areas. Additionally, EES also improves the capabilities of the spinal cord to translate sensory information involving muscle activity regarding standing and walking. The mechanisms of EES involve recruiting the proprioceptive circuits located in the posterior roots of the spinal cord, which thus activates the motor neurons. This led to the emergence of the idea that EES protocols could be developed to target specific areas of the spinal cord. When it comes to movement occurring, motor neurons need to be engaged at the proper time. Thus, spatiotemporal EES would be more effective as compared to empirical stimulation protocols. This has already been shown to be effective in the potency of leg movements in animal models of leg paralysis [28]. When EES is then paired with the overground locomotor training, which is enabled by a gravity-assist device, this apparatus can promote the reorganization of residual neural pathways [28].

Furthermore, on the EES side of neuromodulation, targeted neurotechnologies have been in use for potential functional restoration. There have been developments in creating a wireless environment and performing simulations using hybrid computational models of EES. Each of these models was personalized using MRI and CT scans to estimate the relative recruitment of each posterior root. These simulations would then guide the creation of the optimal electrode configurations. Monopolar pulses were then delivered through EES at increasing intensities. The electrodes that responded the best would then display a projection of muscle response amplitudes. These were depicted as circular dots which then described the spatial selectivity of each electrode. Consequently, if the selectivity of the electrode was insufficient, then the electrical field would be steered using different multipolar electrode configurations [28].

SCS and EES have shown promising results in regard to functional restoration in paraplegic and quadriplegic patients. Spatiotemporal convergence is hypothesized to show neurological recovery with EES. SCS and EES therapies rely heavily on neuroplasticity and would be most effective early on in the rehabilitation process. Intervention closer to the event of the spinal cord injury would maximize the benefits of EES, while SCS could be used after EES has been attempted. EES would also be deemed most effective at the point where the neuromuscular system has not undergone atrophy associated with chronic paralysis. Additionally, improvement in muscle mass and other physiological functions indicates that EES may counteract these deficits [28].

While SCS and EES demonstrate encouraging outcomes, challenges remain. Patient selection criteria, electrode placement, and the variability of individual responses influence the efficacy of SCS [23]. Additionally, the long-term sustainability of benefits and the need for adjustments in stimulation parameters warrant further investigation. Moreover, the cost and accessibility of neuromodulation devices pose practical challenges that need consideration for widespread adoption [23]. The challenges of implementing spinal cord stimulators on a large scale and serving as a mainstream solution for functional restoration still exist. However, it is an option worth pursuing, as, for certain patients, it can provide long-lasting relief.

## 10. Conclusions

Paraplegia and quadriplegia, pervasive consequences of spinal cord injuries, pose significant challenges in regard to functional restoration. The critical time window for surgical interventions within the first 24 h post-injury presents limitations due to safety concerns. The difficulty of performing surgical interventions with a small window of time and minimizing further damage is a current concern. Meanwhile, conventional therapies, including physical interventions, exhibit limited efficacy in achieving comprehensive functional restoration for individuals affected by these conditions. The non-surgical methods are merely attempts at providing satisfaction long-term and are just not feasible solutions for providing the level of functional restoration that is being sought. However, a burgeoning frontier in the field of spinal cord injury treatment is the promising realm of neuromodulation. Neuromodulation, particularly SCS and EES, stands as a frontier in enhancing functional recovery for individuals with paraplegia and quadriplegia post-SCI. While current evidence demonstrates promising results, ongoing research addressing efficacy, optimization, and accessibility is crucial for fully harnessing the potential of neuromodulation in reshaping rehabilitation paradigms and improving outcomes for these individuals. With the current algorithms for optimizing patient selection prove promising, in conjunction with SCS and EES models, the outlook for SCI patients has drastically increased, with more favorable options becoming available. The model for implementing these neuromodulation techniques has improved the outcomes and overall view of recovery post-SCI for many individuals.

This review has illuminated the potential of neuromodulation as a viable therapeutic avenue for addressing the deficits associated with paraplegia and quadriplegia. Notably, SCS has emerged as an alternative intervention, showcasing encouraging signs of functional restoration coupled with heightened patient satisfaction. Studies have underscored the efficacy of SCS in mitigating neuropathic pain and enhancing motor function in individuals with spinal cord injuries, laying the groundwork for its therapeutic potential. These interventions exhibit promise in offering not only pain relief, but also in fostering substantial improvements in motor and sensory functions. Using the patient selection criteria methods and EES, neuromodulation can optimize neuroplasticity to maximize functional restoration, and SCS thus continues the level of functional restoration long after beginning the initial intervention. The limitations of traditional therapies have necessitated the exploration and adoption of innovative strategies such as neuromodulation. Research endeavors have signaled the emergence of neuromodulation, particularly SCS, as an alternative, minimally invasive option in addressing the functional deficits stemming from paraplegia and quadriplegia. While acknowledging the constraints and challenges inherent in the treatment landscape for spinal cord injuries, the integration of neuromodulation, particularly through SCS, presents a transformative paradigm in the pursuit of functional restoration and improved quality of life for affected individuals.
